# Development of Drone-Mounted Multiple Sensing System with Advanced Mobility for In Situ Atmospheric Measurement: A Case Study Focusing on PM_2.5_ Local Distribution

**DOI:** 10.3390/s21144881

**Published:** 2021-07-17

**Authors:** Hirokazu Madokoro, Osamu Kiguchi, Takeshi Nagayoshi, Takashi Chiba, Makoto Inoue, Shun Chiyonobu, Stephanie Nix, Hanwool Woo, Kazuhito Sato

**Affiliations:** 1 Faculty of Software and Information Science, Iwate Prefectural University, Takizawa 020-0693, Japan; 2 Faculty of Systems Science and Technology, Akita Prefectural University, Yurihonjo 015-0055, Japan; snix@akita-pu.ac.jp (S.N.); ksato@akita-pu.ac.jp (K.S.); 3 Faculty of Bioresource Sciences, Akita Prefectural University, Akita 010-0195, Japan; o_kiguchi00120@akita-pu.ac.jp (O.K.); tnaga@akita-pu.ac.jp (T.N.); makoto@akita-pu.ac.jp (M.I.); 4 College of Agriculture, Food and Environment Sciences, Rakuno Gakuen University, Ebetsu 069-0851, Japan; t-chiba@rakuno.ac.jp; 5 Graduate School of International Resource Sciences, Akita University, Akita 010-8502, Japan; chiyo@gipc.akita-u.ac.jp; 6 Institute of Engineering Innovation, Graduate School of Engineering, The University of Tokyo, Tokyo 113-8656, Japan; woo@robot.t.u-tokyo.ac.jp

**Keywords:** PM_2.5_, drone, in situ atmospheric measurement, LoRa, long short-term memory

## Abstract

This study was conducted using a drone with advanced mobility to develop a unified sensor and communication system as a new platform for in situ atmospheric measurements. As a major cause of air pollution, particulate matter (PM) has been attracting attention globally. We developed a small, lightweight, simple, and cost-effective multi-sensor system for multiple measurements of atmospheric phenomena and related environmental information. For in situ local area measurements, we used a long-range wireless communication module with real-time monitoring and visualizing software applications. Moreover, we developed four prototype brackets with optimal assignment of sensors, devices, and a camera for mounting on a drone as a unified system platform. Results of calibration experiments, when compared to data from two upper-grade PM${}_{2.5}$ sensors, demonstrated that our sensor system followed the overall tendencies and changes. We obtained original datasets after conducting flight measurement experiments at three sites with differing surrounding environments. The experimentally obtained prediction results matched regional PM${}_{2.5}$ trends obtained using long short-term memory (LSTM) networks trained using the respective datasets.

## 1. Introduction

Greenhouse gases have increased dramatically in the atmosphere since the Industrial Revolution of the late 18th century. The severity of rising sea levels [1] and abnormal weather changing patterns [2] related to anthropogenic climate change, reflecting the effects of human activities on the climate [3], have been worsening annually for decades. Air pollution has especially been increasing because of diverse and widespread human economic activities and daily life, mainly because of fossil fuel combustion [4]. Air pollution represents a severe difficulty: it not only adversely affects our health in terms of coughing, asthma, and bronchitis, but it also imposes tremendous burdens on the natural environment. As only one of many effects, acid rain and air pollution [5] cause severe damage to crops, soil, lakes, marshes, and forests.

As an important component of air pollution, particulate matter (PM) has been attracting attention worldwide [6]. For such matter, PM${}_{2.5}$ signifies particles having diameter as large as 2.5 μm. Several studies have demonstrated the epidemiological effects of PM${}_{2.5}$ on human health [7,8,9,10,11]. High PM${}_{2.5}$ concentrations increase risk not only of respiratory diseases in terms of asthma and bronchitis but also of cardiovascular disease.

Since the late 1990s, concern about PM${}_{2.5}$ that induces damage to respiratory and circulatory systems has increased rapidly in industrialized countries. The primary and dominant PM${}_{2.5}$ occurrence sources can be classified roughly into two types: natural sources and anthropogenic sources [12]. The widespread combustion of fossil fuels in huge amounts increases greenhouse gases and PM${}_{2.5}$ attributable to anthropogenic sources. To control the increase on a regional level, numerous approaches have been proposed in terms of real-time absorption and emission monitoring, investigation of concentrations, source detection and identification, and specifications of the causative materials.

For real-time monitoring, local governments have provided public measurement and monitoring stations. The PM monitoring values obtained from fixed stations observed at specific intervals are published immediately from online websites. However, installation intervals of typical fixed stations are wide because of initial and maintenance costs. Therefore, land utilization patterns covered by a particular monitoring station differ: residential, cultivating, or industrial areas. Optimization of the appropriate number and placement of fixed stations, such as time and spatial resolution, remains a challenging research task.

As a fundamental property, PM${}_{2.5}$ includes characteristics that affect areas far from the pollution source because of suspension in the atmosphere for long periods. However, the source identification with sufficient accuracy remains a challenging task with respect to temporal and spatial resolution with the current inadequate number of measurement stations, installation intervals, and monitoring frequencies. Detection of PM${}_{2.5}$ sources and their tracking are anticipated not only for scientific elucidation of generation mechanisms but also for administrative measures to alleviate resident concerns. Although meteorological satellites are used primarily for wide-range PM${}_{2.5}$ tracking, the spatial resolution of such data is low. Therefore, improvement of these resolutions is crucially important to elucidate phenomena of high regional PM${}_{2.5}$ concentrations and the actual effects of transboundary pollution.

Herein, filter vibration methods and β-ray absorption methods are generally used for automatic measurement instruments at PM${}_{2.5}$ measurement stations. These instruments present difficulty when moving because they are large and heavy. Moreover, the range of product prices has become a bottleneck for dissemination. With the rise of environmental awareness [13], small, simple, and inexpensive PM${}_{2.5}$ sensors are now commercially available from several electric manufacturers. In 2013, Sharp Corporation introduced a commercially available PM${}_{2.5}$ sensor using a compact optical element with a minimum delay time for measuring [14]. Panasonic Corporation and Omron Corporation, respectively, introduced inexpensive, compact, and highly accurate PM${}_{2.5}$ sensors with light scattering in 2015 and 2018 [15,16]. A compact PM${}_{2.5}$ sensor introduced by Yaguchi Electric Corporation in 2017 has dramatically expanded applications [17] for long-term monitoring with a simple connection for a smartphone. Concomitantly with technological advancements and improvements, PM${}_{2.5}$ sensors and monitoring systems that are small, simple, inexpensive, and highly accurate are expected to be increasingly popular.

We specifically examine a joint research project combining engineering and meteorological sciences for in situ atmospheric measurements using drones [18,19,20,21,22]. For meteorological scientific study, atmospheric measurements have traditionally been conducted using manned aircraft equipped with expensive specialized sensor systems [23]. Another major approach is using balloon sondes combined with inexpensive, lightweight, disposable sensors and radio modules [24]. For engineering and industrial applications, novel approaches [25] and systems [26,27] have been developed to install various sensors on a drone for environmental measurements with advanced mobility.

Particularly, image sensing using drones is expanding the application range [26] in terms of 2D image sensing [28] with RGB cameras, 2.5D image sensing [29] combined with a monomular camera and structure from motion (SfM) technologies, and temperature distribution measurements [30] using a thermal infrared camera. Recently, numerous application studies have been conducted with 3D images obtained using a light detection and ranging (LiDAR) sensor mounted on a drone [31,32,33,34]. Several drone manufacturers provide commercially available drones equipped with modules for real-time image transmission. By contrast, integrated systems are scarce, except for ordinary cameras or LiDAR sensors. Therefore, measurement signals obtained from sensors are usually saved in a storage device mounted on a drone. When using this approach, in situ measurements consisting of real-time data processing and visualization are not available. Measurement signals are processed offline after the drone returns and lands at the home point.

Thanks to the rapid technological evolution of drones, sensors, wireless communication, and tiny onboard computers, in situ measurements are recently becoming active. As a large-scale in situ measurement campaign, the Lower Atmospheric Profiling Studies at Elevation—a Remotely-piloted Aircraft Team Experiment (LAPSE-RATE) [35,36] was conducted in the San Luis Valley of Colorado, USA, from 14 to 20 July 2018. The LAPSE-RATE aimed to improve the understanding of boundary layer structure, cloud and aerosol properties, and surface-atmosphere exchange. Detailed information supporting model evaluation and improvement work was provided by the LAPSE-RATE. The experimentally obtained datasets are publicly available through an archive site co-located with other LAPSE-RATE datasets as part of the community for their project.

For our earlier study [21,22], we developed an in situ carbon dioxide (CO${}_{2}$) measurement system using an industrial drone. We obtained vertical distribution CO${}_{2}$ profiles throughout one year using our proposed measurement system [20]. We compared our obtained profiles with trends obtained by satellite data. However, this system had no communication device to transmit measurement signals from the drone to the ground station. We processed large amounts of measurement signals offline after the respective flights.

This study was conducted to produce a sensor fusion system that can measure not only CO${}_{2}$ but also PM${}_{2.5}$ and atmospheric and environmental information related to air pollution. For measurement targets that change rapidly because of wind and airflows, our novel system includes a remote communication function for the immediate confirmation of measurements and their processing results. The primary objective of this study is to develop a novel system that can use a drone to conduct in situ atmospheric measurements with high maneuverability. We conducted evaluation measurement flight experiments to obtain atmospheric data using our original composite sensor system and remote communication system. Using high-resolution datasets obtained using multiple sensors, this study elucidates regional distribution profiles at several measurement sites having different regional characteristics and surrounding environments. Furthermore, we assess prediction of PM${}_{2.5}$ trends on a regional basis for applying the obtained datasets to a time series feature learning network based on deep-learning technologies.

This paper is structured as follows. In Section 2, we briefly review related studies of atmospheric measurements using a drone and long-range wireless communication systems. Section 3 presents our proposed systems for in situ measurements. Subsequently, Section 4 and Section 5, respectively, present preliminary experiments for wireless communication and sensor calibration. Section 6 presents evaluation experiments of flight measurements at three sites and a distribution prediction produced using a deep-learning-based method. Finally, Section 7 concludes and highlights avenues for future work.

## 2. Related Studies

### 2.1. Drone-Based Atmospheric Measurements

Drones have become explosively popular [37] in recent years because of rapid technological advances and falling costs of brushless motors, lithium-ion rechargeable batteries, high precision sensors, and flight controllers (FCs). Drones are actually categorized as unmanned aerial vehicles (UAVs). Their name derives from drone bees, which have no independent activity in or out of a beehive. Stable lift power is obtained using multiple rotors driven by high-speed motors controlled precisely with an FC. In contrast to a single-rotor helicopter driven by an internal combustion engine, drones fly stably using an FC combined with a global positioning system (GPS) and an inertial measurement unit (IMU). By virtue of their simplicity and versatility, drones are used today in widely diverse applications.

Along with the road map set out in 2018 for the sky industry revolution provided by the Cabinet Secretariat and the Ministry of Economy, Trade and Industry in Japan, drone utilization fields are classifiable into five categories: logistics, disaster rescue, infrastructure maintenance and management, surveying, and agriculture. Drone-based meteorological and atmospheric measurements, which are not directly included in this roadmap, belong to an interdisciplinary field that is closely related to the categories of disaster response and agriculture. However, various challenging hurdles remain in terms of flight duration time, payload, air body size, sensor accuracy, sensor sensitivity, and noise resistance [38].

Juan et al. [39] developed a measurement system using a small drone for automatic creation of CO${}_{2}$ distribution maps used in a greenhouse. Their system comprised a CO${}_{2}$ sensor (MG811; Sandbox Electronics; Finland), a drone (AR.Drone2.0; Parrot SA; Paris, France), and a Raspberry Pi single-board computer for real-time data processing. They demonstrated the creation of a two-dimensional distribution map of CO${}_{2}$ temperature, humidity, and insolation for a 3 m altitude in a greenhouse of 106 × 47 m.

Sasaki et al. [17] developed a drone system to measure the spatial distribution and vertical profiles of SO${}_{2}$ and H${}_{2}$S concentrations in volcanic gases and PM${}_{10}$ and PM${}_{2.5}$ in volcanic ash. The proposed system comprised a commercially available drone (Spider CS-6; Luce Search Co., Ltd., Tokyo, Japan) equipped with a meteorological sensor (150WX; Airmar Technology Corp., Milford, CT, USA), a gas monitor (QARE3; RAE Systems Inc., Sunnyvale, CA, USA), and a PM monitor (Pocket PM${}_{2.5}$ Monitor; Yaguchi Electronics Co., Ltd., Miyagi, Japan). As a preliminary experiment, sensor calibration was performed on a 55 m high meteorological observation tower. For that study, they observed atmospheric pressure, temperature, and humidity along with latitude and longitude as location information. As an in situ observation experiment, they obtained vertical profiles up to an altitude of 1000 m at a site located about 8 km east–southeast of the crater of Mt. Shinnen-dake in Kyushu, Japan. The experimentally obtained results revealed that their drone was able to fly in wind speeds of up to 15 m/s. They demonstrated both the practicality and usefulness of their developed drone system for volcano observation. Moreover, they presented the possibility of estimating wind velocity calculated from the drone altitude.

Rossi et al. [40] developed a drone system to detect gas leaks using a metal-oxide (MOX) sensor. Although no flight altitude was reported, they conducted an evaluation experiment using a drone (S800; SZ DJI Technology Co., Ltd., Shenzhen, China) to detect a gas source, which had been generated intentionally from isopropyl alcohol. Moreover, they simulated the extension of flight duration time for seven drones and eight photovoltaic solar panels.

Villa et al. [41] developed a drone system for monitoring air pollutants and particulate matter. They monitored gases of four types: carbon monoxide (CO), CO${}_{2}$, nitric oxide (NO), and nitrogen dioxide (NO${}_{2}$). Their in situ measurement system comprised a CO${}_{2}$ sensor (SprintIR; Gas Sensing Solutions Ltd., Cumbernauld, UK) and a complex sensor (Alphasense; Gas Sensing Solutions Ltd., Cumbernauld, UK) for three other gases on a drone (S800; SZ DJI Technology Co., Ltd., Shenzhen, China) with a sensor board on an Arduino microprocessor. The experimentally obtained results revealed the influence of downwash on measurements using a pipe to ventilate automobile exhaust gases at a 3 m height.

Ishihara et al. [42] developed a simple drone system with a thermometer (R5011; Hioki E.E. Corp., Nagano, Japan) attached to a drone (Phantom 4; SZ DJI Technology Co., Ltd., Shenzhen, China) for measuring vertical profiles of air temperature and to elucidate mechanisms of mirage occurrence. They conducted a measurement experiment in Hokkaido, Japan. The experimentally obtained results revealed a temperature boundary (thermocline) at a 40–60 m altitude when a mirage occurs. However, the measurement altitude was limited to 100 m because of the drone battery properties when taking measurements at temperatures below freezing.

To observe high-altitude weather, Inoue et al. [43] developed a temperature and humidity measurement system that comprised a drone (Spider CS-6; Luce Search Inc.; Tokyo, Japan), an ultrasonic anemometer (SE-702LM1; FT Technologies Ltd., Sunbury, UK), and a logger (SHTDL-3; Sensirion AG, Staefa, Switzerland). They obtained vertical profiles of wind direction and speed, air temperature, and humidity up to 1000 m over the Sakurajima Volcano in Kagoshima, Japan. To evaluate the consistency of their data, they compared measurement results with standard data obtained from a meteorological measurement tower and a Doppler Lidar.

Chang et al. [44] developed a drone system to measure volatile organic compounds (VOCs) of 106 types, in addition to methane (CH${}_{4}$), CO, and CO${}_{2}$. After they mounted an electroplated stainless steel container with 2 L capacity on a specifically developed large octo-rotor drone, they compared the difference between an air sample obtained at up to 300 m height and a surface air sample obtained at the northernmost tip of Taiwan.

Andersen et al. [45] developed a drone system to observe CO, CH${}_{4}$, and CO${}_{2}$ in the atmosphere. The system, AirCore [46], was composed by the National Oceanic and Atmospheric Administration (NOAA) on a drone (Inspire 1 Pro; SZ DJI Technology Co., Ltd., Shenzhen, China). Using this system, they analyzed the collected air using an analyzer (G2401; Picarro, Inc.; Santa Clara, CA, USA) because AirCore had no attached analytical instrument. They elucidated vertical profiles up to a 500 m height using five flight tests conducted in the Netherlands.

Rüdiger et al. [47] developed a drone system to observe CO${}_{2}$ and sulfur dioxide (SO${}_{2}$) emitted from volcanic eruptions. They composed an original drone prototype using motors (E800; SZ DJI Technology Co., Ltd., Shenzhen, China) and an FC (NAZA M-2; SZ DJI Technology Co., Ltd., Shenzhen, China) with a CO${}_{2}$ sensor (K30 FR; Senseair AB; Delsbo, Sweden) and a SO${}_{2}$ sensor (CiTiceL 3 msT/F; City Technology Ltd., Portsmouth, UK). Using this drone, they conducted in situ measurement experiments at three volcanoes in Italy, Costa Rica, and Nicaragua. Although they assessed and described temporal changes of gases, no altitude was reported.

Weber et al. [48] developed a drone system using a Pixhawk [49] autonomous flight controller to observe traffic-induced pollution plumes around a river bridge. They used a weight optimized optical particle counter (Grimm 1.109; GRIMM Aerosol Technik Ainring GmbH & Co. KG, Ainring, Germany) [50] that can measure PM${}_{10}$, PM${}_{2.5}$, and PM${}_{1}$. Moreover, they mounted an ultrafine particle (UFP) monitor (DiSCmini; Matter Aerosol AG, Wohlen, Switzerland) on their originally developed drone with eight rotors to measure UFPs from 250 nm to 32 μm. They were able to identify traffic-induced air pollution plumes originating from a bridge based on their field experiments of 60 measurement flights.

Wang et al. [51] proposed a drone system to measure PM${}_{2.5}$ and PM${}_{10}$ simultaneously. Their originally developed drone prototype used six motors and a flight controller appropriated from a commercially available drone. They used a digital universal particle concentration sensor (PMS5003ST; Beijing Plantower Co., Ltd., Beijing, China), which measured PM based on the laser-scattering principle. Their conducted flight application tests in vertical and horizontal directions demonstrated the three-dimensional distribution of PM concentration in arid areas. However, the wireless communication range was limited because they used XBeePro for a sensor data communication module.

Okamura et al. [52] measured the CO_2_ horizontal concentration at around a 30 m altitude using a drone (Matrice 100; SZ DJI Technology Co., Ltd., Shenzhen, China) with an on-board CO_2_ measurement device (C2D-W02TR; UDOM Co., Ltd., Mito City, Japan). They obtained original measurement datasets from 35 points at 50 m intervals every 10 s. Results obtained from comparative experiments conducted in an urban residential area revealed that the CO_2_ distribution variation on the flight was less than that on the ground. Their proposed system demonstrated that a stable concentration distribution reflecting the influence of regional components was obtainable after removing effects from variable components.

As a very recent study, Bieber et al. [53] proposed a drone-based aerosol particles sampling impinger/impactor (DAPSI) system for a field study to investigate sources and near-surface transport of biological ice nucleation particles (INPs). The DAPSI system measured PM_10_, PM_2.5_, temperature, relative humidity, and air pressure at 0.5 Hz. They performed a series of sampling missions of 12 flights using two quad-rotor drones (Phantom 4; SZ DJI Technology Co., Ltd., Shenzhen, China) at a remote sampling site near Gosau, Austria. The experimentally obtained results revealed that fluorescence microscopy of impactor foils showed a significant number of auto-fluorescent particles. Moreover, they demonstrated a slight increase in ice nucleation activity of sampled aerosol measured by using a microscopic cooling technique.

### 2.2. Long-Range Wireless Communication for In Situ Measurements

As long-range wireless communication systems, real-time transmission of measurement signals obtained using sensors has various applications in terms of smart farming [54], disaster alerts [55], water monitoring [56], food traceability [57], health diagnosis of social infrastructure such as bridges and tunnels [58], and smart homes [59]. Low-power long-range communication devices are increasingly anticipated for application for various Internet of things (IoT) devices and communication systems [60]. Nevertheless, unresolved technical difficulties and concerns remain in terms of communication failures and interruptions in environments with numerous obstacles, extreme reduction of transmission speed, and communication data loss.

Dambal et al. [61] measured the signal strength of long range (LoRa) wireless communication within and among buildings to investigate factors that affect signal quality in terms of spreading factors and antenna orientation. Moreover, they quantitatively demonstrated the wireless communication performance of a LoRa transmitter mounted on a drone at two altitudes of 25 m and 50 m in urban and suburban areas. Their experimentally obtained results revealed that drone altitudes and antenna orientations are crucially important for improving communications coverage in suburban environments.

Saraereh et al. [62] proposed a LoRa-based wireless communication system for disaster prevention information. Their study was undertaken to provide an alternative method when existing communication networks have been disrupted by disasters such as floods, wildfires, and earthquakes. Their proposed system used a drone as a relay station to improve the message exchange reliability. The results obtained from a simulation-based evaluation demonstrated that LoRa network systems mounted on multiple drones effectively improved packet receiving rates, with only a slight buffer delay.

Chen et al. [63] designed a prototype model of a LoRa-based atmospheric measurement system mounted on a drone. Their study was undertaken not only to transmit sensor signals from remote locations but also to perform sensing tasks using a drone with minimum human resources, burdens, and procedures. They developed a web-based application to store and to visualize measurement signals obtained from drone-mounted air quality sensors. Nevertheless, the main outcomes of their study were limited to simulation results.

### 2.3. Atmospheric Distribution Prediction

Studies have been conducted recently not only to measure the atmospheric environment but also to predict distribution profiles and trends based on machine-learning methods that have been trained using obtained datasets. Masood et al. [64] used a conventional artificial neural network (ANN) and a support vector machine (SVM) to predict PM_2.5_ global distributions. The experimentally obtained results based on a simulation using benchmark datasets obtained from online portals revealed that ANN was superior to SVM for PM_2.5_ prediction accuracy.

Doreswamy et al. [65] assessed PM_2.5_ predictions based on machine-learning algorithms of four representative types using publicly available datasets obtained from 76 air pollution stations over five years. Their experimentally obtained cross-validation (CV) results revealed that gradient boosting regression (GBR) methods achieved the highest accuracy in two evaluation metrics. However, the performance extraction of each algorithm was insufficient because optimizing network parameters were not reported.

Yazdi et al. [66] created a prediction model for daily PM_2.5_ levels using an ensemble machine-learning approach consisting of random forest (RF), gradient boosting machine (GBM), and *k*-nearest neighbor (*k*-NN) algorithms. They conducted an evaluation experiment to predict PM${}_{2.5}$ concentration in 3980 grid cells with a 1×1 km range using long-term datasets obtained for a large and densely populated city over eight years. Although no comparative result was presented for machine-learning algorithms that were combined for ensemble learning, the distributional trends per year in their datasets provided beneficial results in terms of healthcare for urban residents.

Sugiura et al. [67] demonstrated the long-term prediction of PM${}_{2.5}$ distribution using deep recurrent neural networks (DRNN) embedded by dynamic pre-training (DTT): DTT-DRNN. They trained DTT-DRNN using PM${}_{2.5}$ data combined with related data of weather datasets of wind speed and orientation, temperature, sunshine hours, humidity, and rainfall from two-year public datasets of one-hour sampling in 52 Japanese cities. Their experimentally obtained results, when compared to an existing method using a weather model, revealed superior prediction accuracy for urban areas.

Song et al. [68] proposed a residual-learning-based [69] end-to-end deep-learning network model to estimate PM${}_{2.5}$ and PM${}_{10}$ values from scene images obtained using a smartphone camera. First, they calibrated two low-cost portable sensors to provide reliable, highly accurate pollutant measurements. Then, they conducted experiments to demonstrate PM measurements within a distance of up to 500 m, horizontally. After they constructed a comprehensive dataset containing 3024 images based on their calibrated sensors and the empirical experiment, they trained their proposed model using their original image datasets. The experimentally obtained results, when compared to other conventional baselines, revealed that their proposed model outperformed the best deep-learning baseline. They demonstrated that their proposed model can provide air pollutant estimation based on smartphone images or surveillance camera images.

Shang et al. [70] proposed a novel prediction model based on a classification and regression tree (CART) [71] and ensemble extreme learning-machine (EELM) [72] methods to predict hourly PM${}_{2.5}$ concentrations. First, they split datasets by constructing a shallow hierarchical regression tree based on CART. Then, they constructed EELM models using training samples from tree nodes. Moreover, they selected a suitable number of hidden neurons to minimize validation errors. Finally, they compared global and several local EELMs on paths from roots to leaves with the smallest validation errors. They developed original meteorological and air pollutant concentration datasets obtained from a city monitoring center in Yancheng, China. The experimentally obtained results demonstrate that the developed method addresses global–local duality and that it achieved superior accuracy to other machine-learning-based conventional models including RF, support vector regression (SVR), and k-means clustering.

### 2.4. Challenging Tasks and Contributions

As described above, atmospheric environment measurements using small, lightweight, and inexpensive sensors combined with a drone have been taken more actively in recent years. Nevertheless, we consider that three challenging tasks remain for realizing actual applications. The first task is to combine multiple sensors while guaranteeing their accuracy. Currently, the observed particles and components remain limited because of the drone payload. Instead of improving payloads by increasing the drone size, further reductions must be achieved in terms of sensor system size and weight. The downsized sensor characteristics and accuracy can be calibrated precisely by comparison with upper sensors. The second task is to ensure a wireless communication range that allows the maximum measurement flight mobility for a drone. The communication capabilities of conventional drone-based sensor systems have improved in small areas because of the increasing use of Wi-Fi, ZigBee, and Bluetooth protocols. The third task is to predict local distributions from sensor signals obtained using drones for in situ measurements that require flexibility and high mobility. Existing studies of distribution prediction have primarily used datasets obtained from fixed public measurement stations. Moreover, no reported study has addressed distribution prediction combined with fixed stations and drones as mobile and flexible stations. To realize the three tasks presented above, this study was conducted to develop a novel multi-sensor system with a prediction function based on a deep-learning algorithm and a long-range wireless communication protocol.

## 3. Proposed System

### 3.1. Overall System Architecture

**Figure 1:**
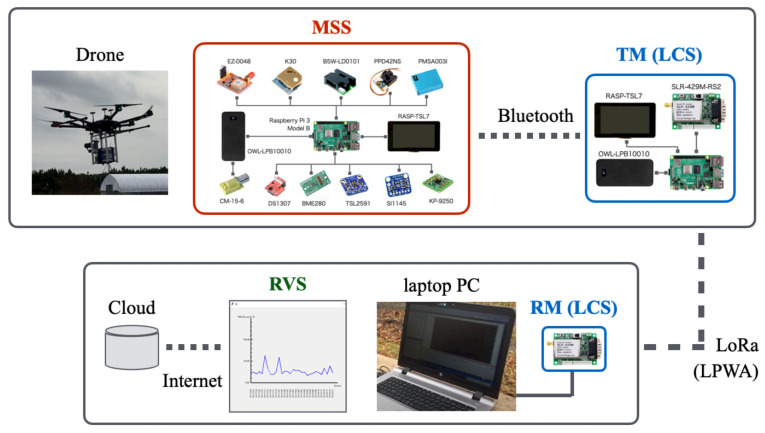
Overall architecture of our proposed system for in situ atmospheric measurements.

Figure 1 depicts the overall architecture of our proposed in situ measurement system. This unified system comprises three subsystems: a multi-sensor subsystem (MSS), a long-range wireless communication subsystem (LCS), and a real-time monitoring and visualizing subsystem (RVS).

An LCS comprises two modules: a transmitter module (TM) and a receiver module (RM). The MSS and TM are mounted on a drone for remotely measuring atmospheric phenomena over a wide area. Together, the MSS and LCS are independent hardware. They communicate using Bluetooth: a short-range, low-power, and low-cost communication protocol [73].

The RM and RVS are installed at a ground station. Using LoRa, which is an attribute of low-power wide-area network (LPWAN) technology, the TM and RM provide long-range wireless communication. Accordingly, the system sends signals measured using the sensors in the MSS to RM using LoRa via the TM using Bluetooth. We connected RM to a laptop computer to visualize measurement results for a dynamic graph immediately, which is the main function of RVS as a part of in situ measurements.

The following describes the details of the MSS, LCM, and RVS development processes including a prototype of our originally developed brackets of four types for the MSS and TM mounted on a drone.

### 3.2. Multiple Sensing of Atmosphere

#### 3.2.1. Specifications

**Figure 2:**
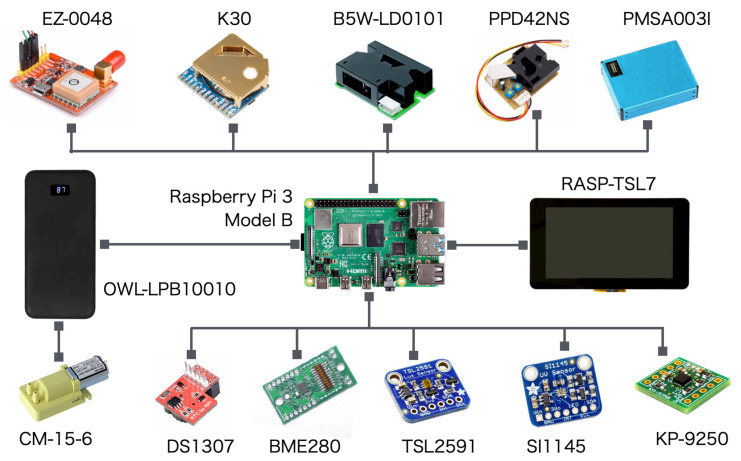
Connection details of respective parts for assembly.

Table 1 presents the listed parts consisting of devices and sensors for developing MSS. Figure 2 depicts connection details of the respective parts.

We compared three commercially available PM sensors: B5W-LD0101 by Omron Corporation, PPD42NS by Shinyei Technology Co., Ltd., and PMSA003I by Beijing Plantower Co., Ltd. These sensors employ a detection method based on the light scattering principle. The minimum detectable particle sizes of LD0101, PPD42NS, and PMSA003I are, respectively, 0.5, 1.0, and 0.3 μm. Table 2 denotes major specifications of three PM sensors from respective datasheets. The basic sensor properties, comparison results, and calibration procedures are described in detail in Venkatraman Jagatha et al. [74].

For CO${}_{2}$ measurements, we used a compact, durable, and reliable sensor (K30; Senseair AB; Delsbo, Sweden) rather than the costly sensor used in our earlier studies [21,22]. This sensor employs the non-dispersive infrared (NDIR) method [75] as the operating principle. As explained herein, the emphasis is on PM${}_{2.5}$ measurements in this case study. We infer that a calibration method must be established because we confirmed large variability for this sensor.

To obtain positional information, we used a built-in GPS sensor (L80-R; Quectel Wireless Solutions Co., Ltd., Shanghai, China), which is a part of the packaged GPS module (L80-R; STMicroelectronics NV; Geneva, Switzerland) specialized for a single board computer (SBC). We connected the L80-R to the SBC using a universal serial bus (USB) cable. In addition to positional information obtained from GPS, we used a real-time clock (RTC) module (DS1307; Adafruit Industries, New York City, NY, USA) to map spatial and temporal information of the measurement signals.

To measure humidity, pressure, and temperature, we used a combined ambient sensor (BME280; Robert Bosch GmbH; Gerlingen City, Germany). Letting *P* and *T*, respectively, denote the air pressure and temperature obtained using the sensor on a drone, then the drone flight altitude $H_{d}$ can be given as

(1) $$H_{d}=\frac{\left(\frac{P_{0}^{-5.257}}{P}-1\right)\times \left(T+273.15\right)}{0.0065},$$

where $P_{0}$ stands for the air pressure at ground level. Herein, the altitudes obtained from the altimeter incorporated in the FC of the drone were used as ground truth (GT) for obtained altitudes calculated from sensor data obtained from the BME280.

We used an ambient lighting sensor (TSL2591; ams AG, Premstaetten, Austria) to obtain supplementary weather information during flight experiments. The illuminance values obtained using this sensor were used as additional information to ascertain weather patterns on a flight measurement day. Moreover, we used an ultraviolet (UV) sensor (SI1145; Adafruit Industries, New York City, NY, USA) to obtain additional weather information. To measure the flight kinematics of the drone, we used an IMU (KP-9250; Kyohritsu Electronic Industry Co., Ltd., Osaka, Japan) that has nine axes: three acceleration axes, three gyro axes, and three geomagnetic axes. The maximum sampling rate was 1 kHz.

To reduce downwash effects from the drone rotors, we used an air pump (CM-15-6; Enomoto Micro Pump Mfg. Co., Ltd., Tokyo, Japan). Similarly to other sensors and devices, the air pump was installed inside the MSS. Therein, the air intake was set to 200 mm from the rotors on the drone top plate. The air intake flow rate was controlled by the air pump motor rotations using a pulse width modulation (PWM) function [76].

For downwash effects, Yang et al. [77] and Wu et al. [78] reported airflow simulation results surrounding rotors. Both simulation results demonstrated that the downwash effects of the rotor bottom were large. By contrast, the downwash on the rotor upper was slight. Therefore, we connected the intake on the upper side and mounted the MSS on the lower side using a silicone rubber tube.

Because of its small size, low cost, and light weight, we used an SBC (Raspberry Pi 3 Model B; Raspberry Pi Foundation; Cambridge, UK) to control sensors and to capture measurement signals in addition to their storage and management. For the connection between the sensors and the SBC, we used USB and general-purpose input–output (GPIO) supported by an inter-integrated circuit (I2C). The operating system (OS) can be selected from several types of Unix distributions. For this study, we installed the official Raspberry Pi OS. Then, we developed applications using C/C++ and Python.

We installed a 7-inch touch screen display (RASP-TSL7; Raspberry Pi Foundation; Cambridge, UK) on the MSS to confirm the operating status of the sensors and devices directly. The screen was operated in sleep mode during a drone flight to reduce power consumption. For the power supply, we used a mobile battery (OWL-LPB10010; Owltech Co., Ltd., Ebina City, Japan). With the addition of this battery, the total weight increased by 326 g. Although the drone used for this study had an external power supply terminal as a standard specification, we mounted an independent battery for the MSS to provide scalability for future extensions. The OWL-LPB10010 capacity is 37 Wh, which can operate all devices and sensors, including the motor pump and the SBC, for approximately five hours.

#### 3.2.2. Assembly

**Figure 3:**
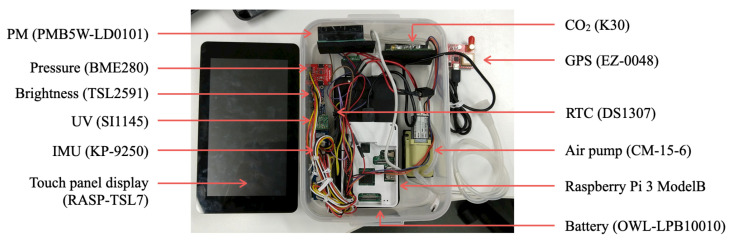
Assembled MSS and part assignment.

Figure 3 portrays the assembled result with the configuration depicted in Figure 2 using the devices and sensors listed in Table 1. We used a polypropylene (PP) box with external dimensions of 172 mm width, 231 mm length, and 78 mm height. We placed rubber sponges on the bottoms of all devices and sensors to fix them in the box and to reduce vibration from motors and rotors.

The total MSS weight is 1.14 kg. We considered the assignment of each component with the center-of-gravity balance while loading the drone. We affixed the battery to the bottom inside the box. A touch screen on the left of Figure 3 was put above all the parts. We put a top cover on the box before mounting it on the drone.

### 3.3. Long-Range Wireless Communication

The important features of LoRa are that it has radio interference robustness, but it has low power consumption and requires no wireless communication license. In recent years, LoRa has been used widely for various communication systems, especially for IoT devices [79]. One LoRa shortcoming is its small communication capacity compared to other communication protocols in terms of Bluetooth, Wi-Fi, and narrow band-IoT (NBIoT) [80]. For IoT devices, the necessity for low-power and long-range communication is more important than the necessity for high-capacity communication [81].

**Figure 4:**
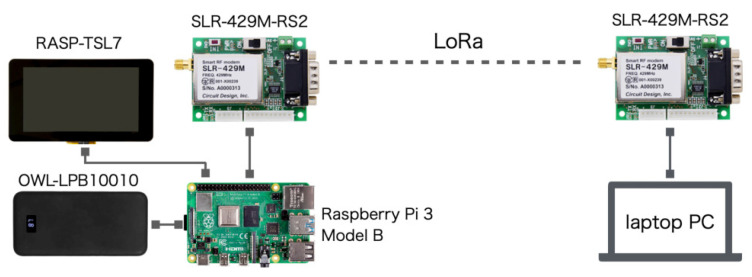
Long-range wireless communication system (LCS) connection architecture.

Figure 4 depicts the LCS connection architecture. We used two LoRa modems (SLR-429M-RS2; Circuit Design, Inc.; Azumino City, Japan). We designed the wired connection between the RM for the ground station side and a laptop computer or a tablet computer. For the drone side, the TM is controlled using the SBC. We used a similar combination of the SBC and the mobile battery for the MSS.

**Figure 5:**
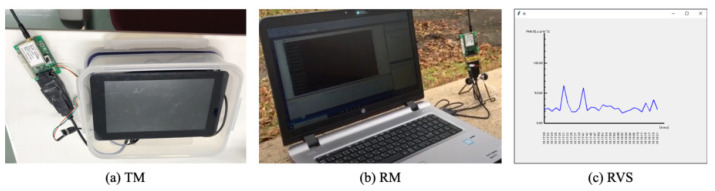
Assembled transmitter module (TM) and receiver module (RM) consisting of LCS.

Figure 5a depicts the TM stored in a PP box, which is the same box as that used for the MSS. After assembly, the TM weight is 1.04 kg including the battery and the touch panel display. Figure 5b depicts the RM connected to a laptop computer.

We used Bluetooth, which is included with Raspberry Pi 3B models as a standard function, to transmit sensor signals from the MSS to LCS. The important benefit of using Bluetooth is not only the increased flexibility and independence of both systems but also the obviation of additional devices and signal cables for communication. The preliminary experiment results revealed that both the MSS and TM stored in independent PP boxes mutually communicated without delay. Moreover, preliminary experiment results obtained for radio interference with other Bluetooth devices revealed that steady communication was achieved in an indoor environment with more than 20 other Bluetooth devices.

### 3.4. Real-Time Monitoring and Visualization

**Figure 6:**
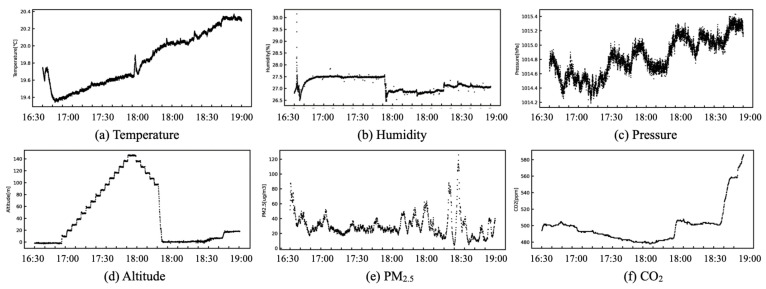
Visualization of measurement signals on real-time visualization system (RVS).

Atmospheric measurement data obtained from the MSS were visualized by the RVS in real time on a computer screen connected to the RM. Figure 5c depicts a graphical user interface (GUI) implemented on the RVS. Figure 6 portrays visualization examples of temperature, humidity, pressure, altitudes, PM${}_{2.5}$, and CO${}_{2}$. The vertical and horizontal axis scales can be set arbitrarily. As a fundamental specification, the visualization signals are updated at 1 Hz. Herein, simultaneous drawing of all measurement signals is unsupported by the RVS because of the LoRa communication capacity.

### 3.5. Drone Mounting

#### 3.5.1. Platform Drone

For this study, we used a large industrial drone (Matrice 600 Pro; SZ DJI Technology Co., Ltd., Shenzhen, China) as a platform to measure atmospheric phenomena using the mounted MSS. Because of its advanced functionality, versatility, and scalability, this drone model has been applied in numerous studies and research projects [82,83,84,85,86,87,88,89,90,91,92] in various application fields. According to the official specifications provided by the manufacturer, the total weight and the maximum payload are, respectively, 10 kg and 6.0 kg. The three-dimensional (3D) body is 1668 mm wide, 1518 mm long, and 727 mm high. Regarding flight performance, its maximum horizontal, ascent, and descent speeds are, respectively, 18 m/s, 5 m/s, and 3 m/s. Onboard batteries of two types are officially provided for this drone. The standard battery set (99.9 Wh × 6 = 599.4 Wh) provides approximately 30 min of flight time, depending on the payload, flight pattern, and wind speed. We used the large capacity battery set (130.0 Wh × 6 = 780.0 Wh), which provides an additional five minutes of flight time.

#### 3.5.2. Originally Developed Sensor Brackets

For mounting the MSS on the drone, we developed four prototype brackets, designated, respectively, as Types 2–5. Table 3 presents the major specifications of Types 1–5. Of these, Type 1 was developed in our earlier studies [21,22].

We set the width and length of Type 2 as equivalent to those of Type 1. These dimensions were determined by the dimensional constraints for the brackets on the drone bottom side. Although the heights of Types 1 and 2 were equivalent, the MSS was mounted alone on the drone because of the volume. Therefore, Type 2 was limited to use without the LCS.

For modification from Type 2 to Type 3, the height was enlarged from 190 mm to 350 mm, although the width and length of Type 2 were retained. This modification accommodated the mounting of both the MSS and LCS on the drone. Moreover, we mounted a monocular camera for non-viewed flight combined with a first-person view (FPV) function. However, the center of gravity of the payload was farther from that of the main drone body. This gap decreased the in-flight stability. To avoid interference with the camera, Type 4 was designed to accommodate mounting of both the MSS and LCS. Using offset parts, the dimensions of Type 4 were enlarged by 20 mm width and 55 mm length compared to those of Type 3. Although the center of gravity of this bracket was close that of the drone, the total weight was increased from 1.24 kg to 1.83 kg.

**Figure 7:**
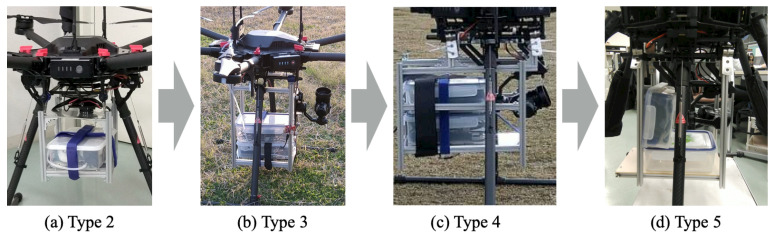
Appearance of Types 2–5 after assembly.

The Type 5 width, length, and height were resized to the equivalent specification lengths as Type 3. For this modification, the LCS installation was changed from horizontal to vertical. This change avoided interference with the camera. The weight of Type 5 was 0.25 kg less than that of Type 3 and 0.84 kg less than that of Type 4. Figure 7 depicts the appearances of Types 2–5 after assembly.

For our earlier study, Type 1 was attached to the carbon poles under the drone main body with joint parts manufactured using a 3D printer. The four pillars made of hollow aluminum frames and the two panels made of carbon plates prevented efficiency and increased the total manufacturing time. Moreover, the excessive use of bolts and nuts reduced the work efficiency of installation and maintenance considerably. Therefore, for this study, we changed the joint parts molded using a 3D printer to commercially available clamps. The carbon plates were replaced with acrylic plates. Although the increased weight was a shortcoming, manufacturing was simple. The camera was installed to fly at altitudes over 150 m, but the Japanese Civil Aeronautics Law restricts the maximum altitude for drone flight to 150 m. We installed a monocular camera for a visibly monitored flight, which became possible with flight permission deregulation from the Ministry of Land, Infrastructure, and Transport of Japan.

The developed brackets were installed on the carbon pipes under the drone via sponge rubber to counter vibration transmitted from the high-speed rotating motors. Therefore, installation of Types 2–4 is easier than that of Type 1 because of the improvement of the clamps that were used. The payloads of Types 2–5 are smaller than that of Type 1 because of the expanded size. Considering a 6.0 kg payload, the drone can accommodate 5.04 kg as its maximum payload and 4.17 kg as the minimum payload.

## 4. Communication Experiment

As a preliminary experiment before conducting measurements using the MSS, we evaluated the LCS communication performance.

### 4.1. Ground Communication Experiment

#### 4.1.1. Setup

**Figure 8:**
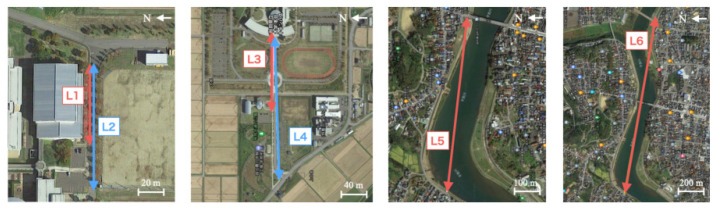
Ground communication experiment of six sections.

This experiment was conducted at six sections, labeled as L1–L6, on the ground without using a drone. Figure 8 depicts the respective sections and their surrounding environments. Communication experiments on L1–L4 were conducted at the Honjo campus (39°39′35″ N, 140°7′33″ E) of Akita Prefectural University, Yurihonjo city, Japan. Herein, L1 and L3, respectively, represent parts of L2 and L4. The experiments of L5 and L6 were conducted near a river that runs near the campus. Table 4 presents dates and distances in each location.

#### 4.1.2. Results

Table 5 denotes experimentally obtained results. We set the data transmission period from the TM to RM to 1 Hz. Here, the RM rejected data that could not be received within 1 s. Let $N_{D}$ and $N_{R}$, respectively, represent the number of transmission data and the number of receiving data. As evaluation criteria, communication accuracy *A* is defined as the following equation:

(2) $$A=\frac{N_{R}}{N_{D}}\times 100.$$

The L1–L4 results up to a distance of 490 m were achieved with no data reception failure. The experimental site, which is located at the university campus surrounded by rice fields, was chosen with the expectation of yielding meaningful results. This area has no particular obstacle other than street trees. The accuracies of L5 and L6 were, respectively, 98.0% and 88.3%. At L5, the TM was visible from the RM. By contrast, it was invisible at L6. For this distance gap of approximately 500 m, the accuracy difference was 9.7%. An experiment in the town area might be affected by radiowave interference. The reception error was improved by changing the direction of the whip antenna used for the RM. However, we assumed that the accuracy would have been lower if both the TM and RM were located in a downtown area.

### 4.2. Flight Communication Experiment

#### 4.2.1. Setup

**Figure 9:**
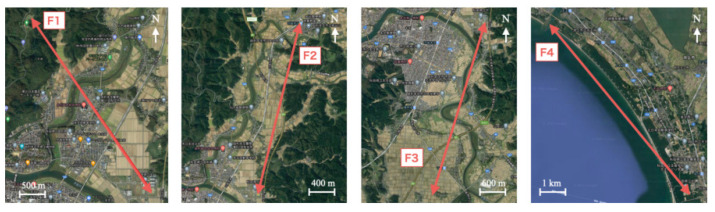
Flight communication experiments of four sections.

This experiment was conducted in four sections, labeled as F1–F4. We mounted the TM on the drone as presented in Figure 1. Figure 9 depicts the respective sections and their surrounding environment. Communication experiments on F1–F3 were conducted between the Honjo campus as the transmission point from the drone and other receiving points on the ground in the suburbs. F4 was conducted along the coastline between the Akita campus (39°80′12″ N, 140°04′62″ E), Akita Prefectural University, Akita City, Japan, as the transmission point and the receiving point at a beach. F1 has a 100 m elevation difference. By contrast, F2–F5 has no elevation difference. Table 6 presents the dates and distances of the respective locations.

During the respective experiments, the drone flew up to 150 m, the limit allowed by the Japanese Civil Aeronautics Law. To change the altitude, the drone moved vertically from the takeoff point. We did not move the drone horizontally.

#### 4.2.2. Results

Table 7 presents experimentally obtained results. We set similar evaluation criteria to those used for ground communication experiments.

The accuracy of F1, which is the shortest distance with the elevation difference, is the second-highest accuracy next to that of F4. Although the gap distance between F2 and F3 is 100 m, the accuracy of F2 is lower than that of F3. We infer that F2 had effects from the highway between the two points. By contrast, F3, which has only a river and rice fields between the two points, demonstrated the realization of stable communication. The F4 with the longest distance exhibited the highest accuracy. This trend is probably attributable to the fact that most areas between the two sites are occupied by the seashore. Herein, for all the experiments of F1–F4, communication was not possible when the drone was on the ground. This experiment revealed that remote communication using LoRa was achievable with the altitude for the TM provided by the drone.

## 5. Preliminary Sensor Comparison Experiment

We verified dust sensors of three types for measuring PM${}_{2.5}$: the primary measurement and monitoring target for this study.

### 5.1. Experiment Setup and Sensor Comparison

**Figure 10:**
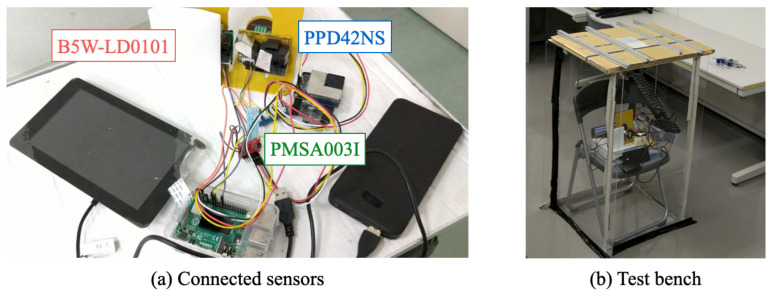
Sensor comparison experiment.

The comparison target sensors were B5W-LD0101, PPD42NS, and PMSA003I as shown in Table 1. Figure 10a depicts the wired connections of these sensors. The SBC connected to respective sensors obtained measurement signals simultaneously. As a temporal test bench, we prepared a simplified chamber separated by acrylic plates, as shown in Figure 10b. The inside dimensions are 500 mm long, 500 mm wide, and 1000 mm high. The top part is covered by wood boards. We installed sensors at 400 mm from the floor.

**Figure 11:**
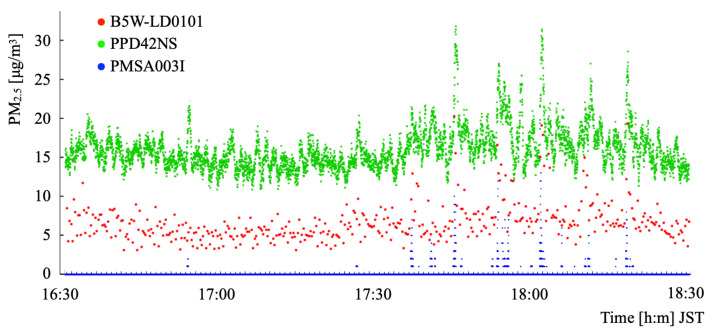
Time-series changes of PM${}_{2.5}$ from three sensors.

Measurements were conducted for two hours. The top cover was closed during the first half (16:30–17:30 JST) of the period. Subsequently, the top cover was opened during the second half (17:30–18:30 JST). We allowed dust into the test bench intentionally. Figure 11 depicts changes of time-series PM${}_{2.5}$ data obtained from the respective sensors.

The sensor output characteristics from PPD42NS are high. Those from PMSA003I are low. The output characteristics from B5W-LD0101 are intermediate between them. The output signals from PPD42NS and B5W-LD0101 in the first half-hour showed a range within ±5μg/m${}^{3}$ variation. The signal outputs in the last half-hour showed pronounced responses from all the sensors at the time when the top cover was opened for adding dust. The experimentally obtained results demonstrated that each sensor had similar response characteristics with different offset values.

### 5.2. Calibration with AEROS

#### 5.2.1. Setup

We selected B5W-LD0101 as the target sensor evaluated in this experiment. As the GT for this experiment, we used the public PM${}_{2.5}$ data provided by the atmospheric environmental regional observation system (AEROS) of the Ministry of the Environment of Japan. This experiment was conducted at the Honjo monitoring station (39°38′43″ N, 140°05′18″ E) provided by a local government. The monitoring data transmitted to AEROS using public lines can be accessed immediately on a website.

**Figure 12:**
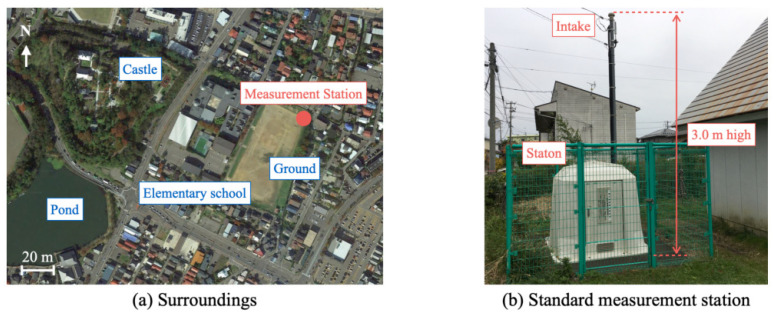
Honjo monitoring station for AEROS.

Figure 12 depicts the appearance of the standard monitoring station and its surroundings. This station is located in the city center at a corner of an elementary school. The area is surrounded by houses with no tall buildings. The measurement instrument, made of fiber reinforced plastics (FRP), is stored in the outer walls. The air intake is provided at 3 m from the ground.

Table 8 shows the weather conditions on the respective measurement days. It was cloudy with no rainfall or sunshine, 15 °C, 72% humidity, and south–southwest 3.3 m/s winds.

#### 5.2.2. Results

**Figure 13:**
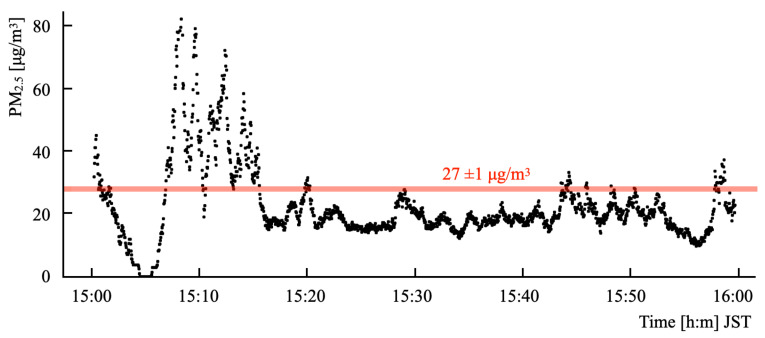
Time-series output values from B5W-LD0101.

Figure 13 depicts time-series sensor output values. For the first 15 min, the output values were unsteady with wide fluctuations. Subsequently, the output values stabilized at around 20 μg/m${}^{3}$. The one-hour mean value from the Honjo monitoring station during this period was 27 ± 1 μg/m${}^{3}$. The one-hour mean value obtained using our system was 24 μg/m${}^{3}$. Comparison of both values shows the difference as 3 μg/m${}^{3}$.

### 5.3. Calibration with IPM2.5-NA

#### 5.3.1. Setup

This experiment was conducted for sensor calibration while maintaining a sufficient measurement period for time resolution. For this calibration, we used an indoor air quality monitor (AirAssure IPM2.5-NA; TSI Inc.; Shoreview City, MN, USA) as a benchmark device. Herein, IPM2.5-NA is certified by the United States Environmental Protection Agency as the standard method for tapered element oscillating microbalance (TEOM) [93]. It has been certified by the Japanese Ministry of the Environment as the PM${}_{2.5}$ automatic measurement certification equivalent device. Table 9 presents the main specifications of IPM2.5-NA.

**Figure 14:**
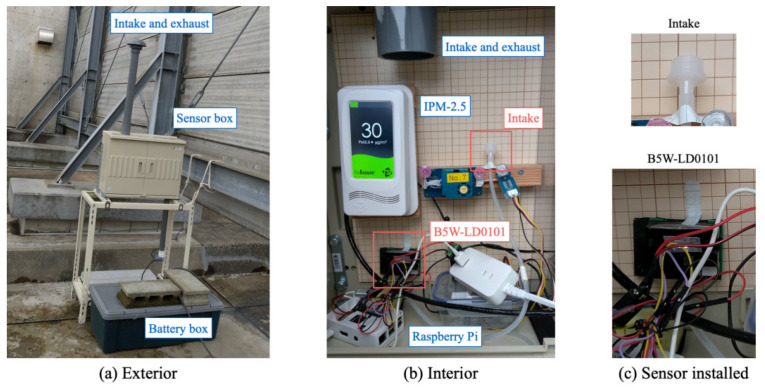
Originally developed benchmark measurement station installed on a three-story building roof for sensor calibration using IPM2.5-NA.

We originally developed a benchmark measurement station. Figure 14a depicts the exterior of the metal shielding box containing the parts and instruments. This experiment was conducted by installing them on a three-story building roof. The air intake and exhaust ports were located at 2 m above the ground. Figure 14b depicts the layout inside the box.

#### 5.3.2. Results

**Figure 15:**
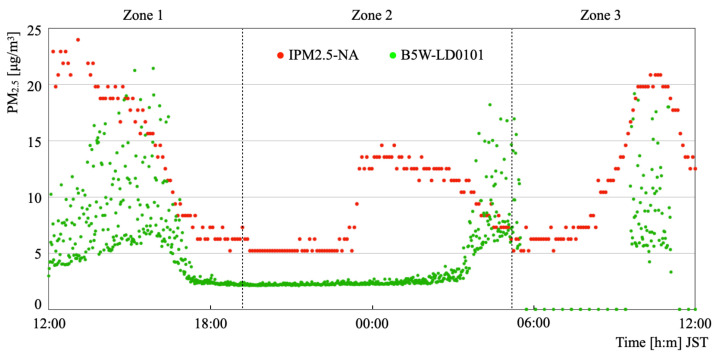
Output signals obtained during 24 h.

Figure 15 depicts the results of comparison of the measurement data obtained during the sequential period of 24 h on 22–23 January 2021. The weather conditions on the first day were 3.4 °C mean temperature, 81% mean humidity, 3.2 m/s mean wind speed, and 1016.4 hPa mean pressure. The weather conditions on the second day were 0.8 °C mean temperature, 66% mean humidity, 2.4 m/s mean wind speed, and 1022.3 hPa mean pressure. The weather was a typical winter pattern for the Sea of Japan side, with weather being cloudy and sometimes sunny on both days. The overall trend indicated that the measured values from B5W-LD0101 were lower than those from IPM2.5-NA.

**Figure 16:**
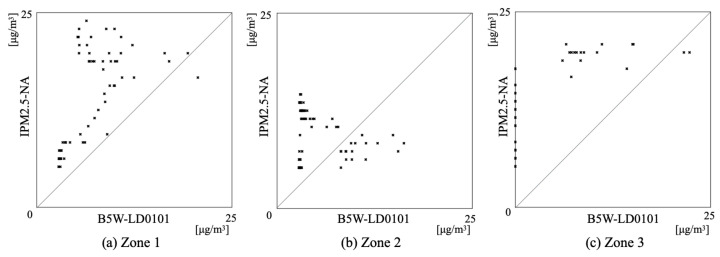
Scatter plots between output signals of IPM2.5-NA and B5W-LD0101 in the respective zones.

During the 24 h, the IPM2.5-NA output signals exhibited three noticeable changes. We divided the time-series signal changes into three zones to observe details of the data distribution. Figure 16 depicts scatter plots for the respective distributed data of Zones 1–3. The output signals from B5W-LD0101 showed a lower trend than those from IPM2.5-NA. Comparison results for each zone indicated that Zone 1 exhibited the highest correspondence. Although the B5W-LD0101 output signals followed these changes, the changes of Zones 2–3 occurred with a time delay. Regarding the offsets of both sensors, the trend of PM${}_{2.5}$ concentration changes can be captured adequately by B5W-LD0101. We consider that B5W-LD0101 can measure time-series concentration changes equivalently to IPM2.5-NA with a greater temporal resolution with maintaining appropriate accuracy compared to the monitoring station if we apply the results described above as a calibration.

## 6. Application Experiments for Flight Measurement and Distribution Prediction

**Figure 17:**
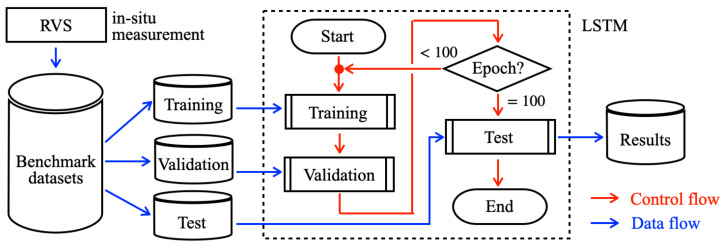
Flowchart of our proposed prediction method using LSTM.

The application experiments are conducted to achieve two objectives. The first objective is the use of our proposed system for flight measurement experiments. The second objective is to evaluate distribution prediction accuracy using a method based on a deep-learning model from obtained sensor signals. Figure 17 depicts the flowchart of our proposed prediction system using long short-term memory (LSTM) [94].

### 6.1. LSTM

**Figure 18:**
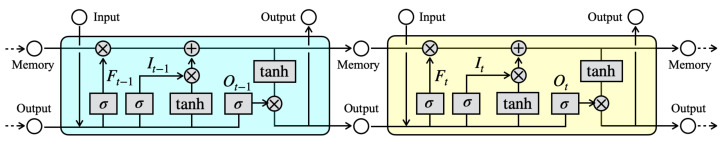
LSTM network architecture of the hidden layer. Signals progress from left to right.

As a predictive model, LSTM is an improved recurrent neural network (RNN) architecture that solves the vanishing gradient problem [95]. LSTM and its derivative models have been used widely in existing studies to forecast typhoon formation and hourly air pollution [96], to analyze meteorological sensor signals [97], and to estimate PM${}_{2.5}$ concentrations [68,98]. Figure 18 portrays a typical LSTM network architecture of hidden layers.

The internal LSTM structure comprises hidden layer units with memory cells that are called LSTM blocks and three types of gates: input gates, forgetting gates, and output gates. The vanishing gradient problem [95] is accomplished by this mechanism. Input gates select enabled or disabled input feature signals. Forgetting gates select the permission to reset internal information stored in cells. Output gates determine the amount of information that is transmitted at the next phase. Based on RNN algorithms, LSTM networks provide one-step later prediction that is conducted from the input feature signals at the current time *t* and the feedback signals to the hidden layer at the previous time t−1. The memory cells save internal information for a long period, which provides an important benefit compared to RNNs for modeling temporally distant dependencies.

Letting $x_{t}$ and $c_{t}$, respectively, represent the input feature signal and output from memory cells, and letting $I_{t}$, $F_{t}$, and $O_{t}$, respectively, denote the outputs of the input, forgetting, and output gates, then LSTM output $H_{t}$ is obtained as shown below:

(3) $$I_{t}=\sigma \left(W_{i}x_{t}+R_{i}h_{t-1}+b_{i}\right),$$

(4) $$F_{t}=\sigma \left(W_{f}x_{t}+R_{f}h_{t-1}+b_{f}\right),$$

(5) $$O_{t}=\sigma \left(W_{o}x_{t}+R_{o}h_{t-1}+b_{o}\right),$$

(6) $$c_{t}=c_{t-1}⊗f_{t}+i_{t}⊗\tanh\left(W_{z}x_{t}+R_{z}h_{t-1}+b_{z}\right),$$

(7) $$H_{t}=o_{t}⊗\tanh\left(c_{t}\right)$$

where $W_{i,f,o,z}$, $R_{i,f,o,z}$, and $B_{i,f,o,z}$, respectively, denote input weights, recurrent weights, and biases. Moreover, σ and ⊗, respectively, denote the sigmoid function and the element-wise product.

The dominant role of input gates is to update cell states. The forgetting gates control propagation signals to refer to a previous cell state $c_{t-1}$. Moreover, unnecessary signals are removed by the forgetting gates to prevent excessive information from the earlier cell output split by short-term and long-term memories. The output gates control update values from hidden units. Similar to the input gates, the output gates have a mechanism to avoid inappropriate weight updates for redundant and undesired signals. In addition, the current cell state $c_{t}$ and the LSTM output block $h_{t}$ are also used to calculate the subsequent input data at t+1. As a remarkable characteristic, LSTM networks have a dynamic adjustment mechanism that provides the previous and forward signals while maintaining $c_{t}$ in addition to $H_{t}$.

### 6.2. Measurement Flight Experiment Results

**Figure 19:**
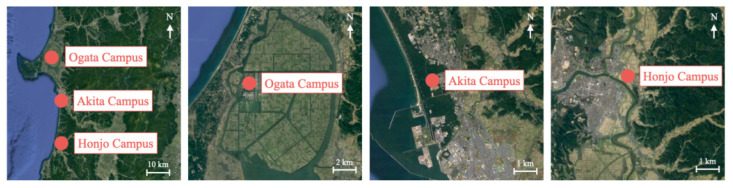
Locations of the respective campuses and their surroundings.

For this experiment, we conducted flight measurement experiments during the period and at sites shown in Table 10. All sites were on the Akita Prefectural University campus. Figure 19 depicts aerial photographs with the locations of the respective campuses and their surroundings. Ogata campus (40°00′64″ N, 139°95′54″ E) is located in a village on a reclaimed brackish lake. The campus is surrounded by rice fields. Akita Campus is located near the coast, surrounded by pine forests. Honjo Campus is located in a suburb surrounded by rice fields and forests. We obtained three datasets, labeled as D1–D3, at each site.

**Figure 20:**
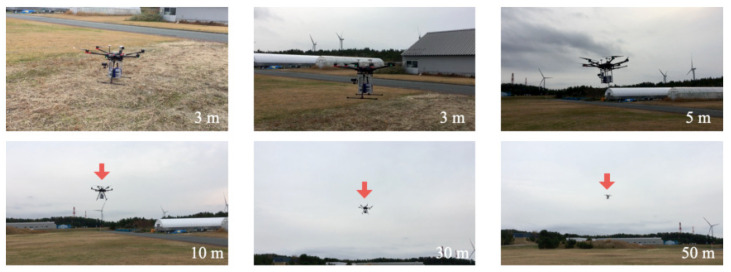
Photograph of D2 flight experiment. The numbers in the lower right corner of respective photographs indicate the approximate distances between the photographer and the drone.

Figure 20 depicts flight experiment photographs at Akita Campus when we obtained D2. We operated the drone manually using an automatic position adjustment function as a program mode. Using a set of large-capacity batteries, the flight time was approximately 30 m with this payload. For this flight experiment, the LCS transmitted PM${}_{2.5}$ measurement signals from the TM to RM without delay.

**Figure 21:**
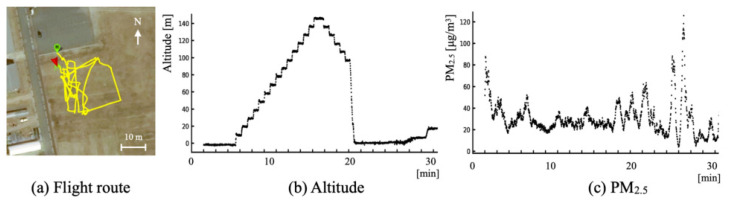
Flight measurement example of obtained signals from three key sensors.

Figure 21 depicts an example of obtained measurement signals from three key sensors: flight route data obtained using GPS; altitude data obtained using the humidity and pressure sensor, and calculated using Equation (1); and time-series data of changes in PM${}_{2.5}$. The data sampling frequency was 1 Hz.

### 6.3. PM${}_{2.5}$ Concentration Prediction Results

Table 11 presents the major LSTM parameters. The numbers of epochs, batch sizes, and intermediate layers units were set, respectively, to 100 generations, 2, and 50 units [99]. Herein, the batch size defines the group number of training samples that propagate through the network inside. The batch size allows for a more refined search to the best local optima [100]. Subsequently, for a stochastic gradient descent (SGD) [101] optimization algorithm, we used RMSprop [102] based on the studies by Xu et al. [103] and by Zou et al. [104]. Let *L* be a look-back parameter, which is one meta-parameter used to refer to training histories. Moreover, *L* represents the number of previous time steps to be considered as input [105]. For training and prediction, we evaluated *L* by changing it to three steps: 30, 10, and 5.

As evaluation criteria of this study, we used the root mean squared error $E_{RMS}$ as defined by the following equation:

(8) $$E_{RMS}\left(y,\hat{y}\right)=\sqrt{\frac{1}{n}\sum_{i=0}^{n-1}{\left(y_{i}-{\hat{y}}_{i}\right)}^{2}},$$

where $y_{i}$, ${\hat{y}}_{i}$, and *n*, respectively, represent the true, predicted, and total number of output signals. The data splitting ratio was 2:1 for training and testing. For the training phase, 20% of the training data was used for validation. Table 12 presents experimentally obtained results.

As an overall tendency, D2 exhibited the lowest $E_{RMS}$ among the three datasets. The optimum *L* parameter value for D2 was found to be 5. For D1 and D3, the *L* parameter optimum values differed in training and testing. The range of variation in testing was greater than that in training. These results indicate that an optimal value search using computational costs is a relevant factor in prediction accuracy for a task that is unnecessary for real-time processing.

**Figure 22:**
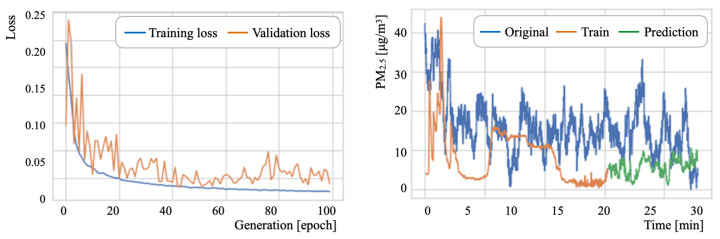
Loss curves and PM${}_{2.5}$ prediction results for D1.

**Figure 23:**
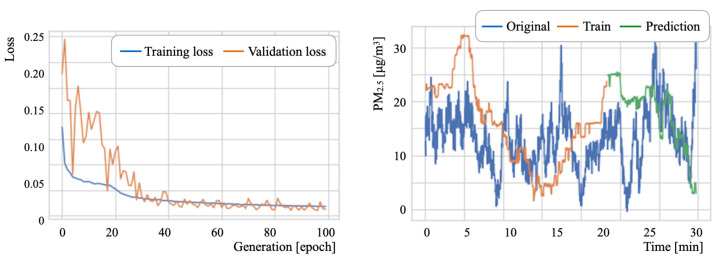
Loss curves and PM${}_{2.5}$ prediction results for D2.

**Figure 24:**
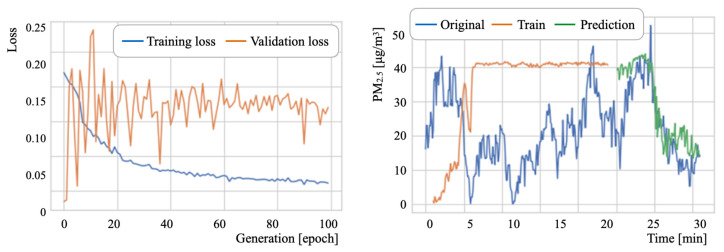
Loss curves and PM${}_{2.5}$ prediction results for D3.

As details of the prediction experiment results, Figure 22, Figure 23 and Figure 24 depict the respective loss curves and the prediction results for test datasets. The loss values decreased concomitantly with the training progress, except for the D3 validation loss. For the D2 tendency, the validation loss converged to a similar value to that of the training loss. For the D3 tendency, the validation loss remained around 0.15. By contrast, the amplitude of loss values decreased as the learning progressed. Although detailed changes of the source data were unmatched to the prediction results, the prediction curves showed an approximation of the global tendency.

Finally, as an example for comparison, the mean $E_{RMS}$ for 10 trials with the DTT-DRNNs proposed by Sugiura et al. [67] was 6.92. Moreover, the mean $E_{RMS}$ with the ResNet-LSTM model proposed by Song et al. [68] was 6.56. Furthermore, the mean $E_{RMS}$ with the CART-EELM method proposed by Shang et al. [70] was 8.96. Although the benchmark datasets differ between our method and these existing methods, our obtained $E_{RMS}$ is lower than their results.

## 7. Conclusions

This study was conducted using a drone with advanced mobility to develop a unified sensor and communication system as a new platform for in situ atmospheric measurements. We developed a multi-sensor system with features of small size, light weight, simplicity, and cost-effectiveness for multiple measurements of atmospheres and for obtaining their related environmental information. Moreover, we developed a long-range wireless communication system and a real-time monitoring and visualizing system for in situ local area measurements. The experimentally obtained results demonstrated that the mean communication accuracies were 97.4% up to 1360 m for ground communication cases and 85.3% up to 13,000 m for flight communication cases. Furthermore, we developed four prototype brackets with optimized assignments of sensors, devices, and a camera for mounting on a drone as a unified system platform. Results of calibration experiments including comparison to two upper-grade standard PM${}_{2.5}$ sensors demonstrated that our sensor system follows the overall tendencies and changes. We obtained original datasets from flight measurement experiments conducted at three sites of different surrounding environments. The experimentally obtained prediction results indicated regional PM${}_{2.5}$ trends using LSTM trained using the respective datasets. As a case study focusing on PM${}_{2.5}$ local distribution prediction, we obtained 2.60, 1.97, and 14.74 μg/m${}^{3}$ for the lowest $E_{RMS}$.

For our future work, based on periodic flight measurements, we would like to verify the durability of our proposed system and its stability for long-term operation. We also would like to increase measurements to be stepped up at other sites to achieve more robust forecast results. We intend to append sensors to elucidate the relation between the payload and flight times of various drone types. Moreover, we expect to develop several sets of our proposed system for conducting simultaneous flight measurements to improve spatial and temporal resolutions. Furthermore, we must verify long-term PM${}_{2.5}$ distribution properties related to regional and seasonal changes.

## Figures and Tables

**Table 1:** Devices and sensors for developing MSS.

Target	Model Name	Manufacturer
PM	B5W-LD0101	Omron Corporation, Kyoto, Japan
PM	PPD42NS	Shinyei Technology Co., Ltd., Kobe, Japan
PM	PMSA003I	Beijing Plantower Co., Ltd., Beijing, China
CO${}_{2}$	K30	Senseair AB, Delsbo Sweden
GPS	L80-R (SKU:EZ-0048)	Quectel Wireless Solutions Co., Ltd., Shanghai, China
Humidity and pressure	BME280	Robert Bosch GmbH, Stuttgart, Germany
Ambient light	TSL2591	ams AG, Premstätten, Austria
UV	SI1145	Adafruit Industries, New York, NY, USA
IMU	KP-9250	Kyohritsu Electronic Industry Co., Ltd., Osaka, Japan
RTC	DS1307	Adafruit Industries, New York, NY, USA
Air pump	CM-15-6	Enomoto Micro Pump Mfg. Co., Ltd., Tokyo, Japan
SBC	Raspberry Pi 3 Model B	Raspberry Pi Foundation, Cambridge, UK
Touch panel display	RASP-TSL7	Raspberry Pi Foundation, Cambridge, UK
Battery	OWL-LPB10010	Owltech Co., Ltd., Kyoto City, Japan

**Table 2:** Major specifications of three PM sensors.

Parameter	B5W-LD0101	PPD42NS	PMSA003I
Manufacture	Omron	Shinyei Technology	Beijing Plantower
Sensor Type	Light scattering photometer		
Detectable size range	0.5 μm	1.0 μm	0.3 μm
Size (H × W × D)	52 × 39 × 18 mm	59 × 42 × 22 mm	51 × 36 × 14 mm
Weight	20 g	20 g	28 g

**Table 3:** Major specifications of originally developed sensor brackets of four types.

Type	Wide [mm]	Long [mm]	High [mm]	Weight [kg]	Camera
1	160	235	195	0.45	unmount
2	160	235	190	0.94	unmount
3	160	235	350	1.24	mount
4	180	290	270	1.83	mount
5	160	235	350	0.99	mount

**Table 4:** Experiment dates and distances of respective sections.

Parameter	L1	L2	L3	L4	L5	L6
Distance [m]	60	90	250	490	860	1360
Data	20 July 2020		30 July 2020		17 September 2020	
Weather	Sunny		Sunny		Sunny	
Atmospheric pressure [hPa]	1008.8		1011.8		1007.2	
Temperature [°C]	28.5		27.4		27.9	
Humidity [%]	59		67		63	
Wind speed [m/s]	4.9		5.4		3.3	
Wind direction	WSW		WSW		SSE	

**Table 5:** Ground communication experiment results.

Index	$N_{D}$	$N_{R}$	*A* [%]
L1	30	30	100
L2	30	30	100
L3	30	30	100
L4	100	100	100
L5	100	98	98.0
L6	60	53	88.3
All	350	341	97.4

**Table 6:** Experiment dates, distances, and meteorology information of respective sections.

Parameter	F1	F2	F3	F4
Date	9 October 2020	22 October 2020	6 November 2020	13 November 2020
Distance [m]	3500	5700	5600	13,000
Weather	Sunny	Sunny	Sunny	Sunny
Atmospheric pressure [hPa]	1023.4	1013.3	1018.5	1019.4
Temperature [°C]	18.5	20.2	16.3	13.8
Humidity [%]	52	57	75	65
Wind speed [m/s]	2.4	5.2	3.5	2.4
Wind direction	NNE	ESE	S	ESE
Flight altitude [m]	≤150	≤150	≤150	≤150

**Table 7:** Flight communication experiment results.

Index	$N_{D}$	$N_{R}$	*A* [%]
F1	347	309	89.0
F2	190	135	71.1
F3	390	339	86.9
F4	87	82	94.3
All	1014	865	85.3

**Table 8:** Weather conditions.

Parameter	Value
Date	6 November 2020
Time (JST)	15:00–16:00
Weather	Cloudy
Atmospheric pressure	1019.5 hPa
Temperature	15.3 °C
Humidity	72%
Wind speed	3.3 m/s
Wind direction	SSW
Precipitation	0 mm
Hours of sunshine	0 h

**Table 9:** Major specifications of IPM2.5-NA.

Parameter	Specification
Sensor Type	Light scattering photometer
Aerosol concentration range	5–300 μg/m${}^{3}$
Zero stability	±10 μg/m${}^{3}$
Time constant	5 min. trailing average
Screen update frequency	1 Hz
Screen resolution	1 μg/m${}^{3}$
Size	H 162 × W 85 × D 33 mm
Weight	200 g

**Table 10:** Details of flight measurement experiment conditions.

Parameter	D1	D2	D3
Date	16 October 2020	13 December 2020	18 December 2020
Time (JST)	13:30–14:39	15:10–15:53	15:17–16:03
Latitude	39°39′12″ N	39°80′12″ N	40°00′64″ N
Longitude	140°04′62″ E	140°04′62″ E	139°95′54″ E
Site name	Honjo Campus	Akita Campus	Ogata Campus
Weather	Sunny	Rain	Sunny
Atmospheric pressure [hPa]	1019.4	1018.7	1019.6
Temperature [°C]	14.3	11.8	12.3
Humidity [%]	48	91	67
Wind speed [m/s]	1.1	1.7	2.9
Wind direction	ENE	ENE	SE
Flight altitude [m]	≤150	≤150	≤150

**Table 11:** Major LSTM parameters and their setting values.

Parameters	Setting Values
Learning iteration [epoch]	100
Batch size	2
Validation rate	0.2
Number of hidden layers	50
Optimization algorithms	RMSprop
*L*	30, 10, and 5

**Table 12:** $E_{RMS}$ for training and test datasets. Bold underlined values are minimum values.

Dataset	*L*	Training [μg/m${}^{3}$]	Test [μg/m${}^{3}$]
D1	30	3.06	3.99
	10	**1.80**	3.73
	5	2.01	**2.60**
D2	30	3.07	9.57
	10	1.97	4.48
	5	**1.59**	**1.97**
D3	30	6.01	**14.74**
	10	**5.84**	16.23
	5	6.12	19.07

## Data Availability

Datasets described as a result of this study are available on request to the corresponding author.

## Abbreviations

The following abbreviations are used in this manuscript:

ANN	Artificial neural network
AEROS	Atmospheric environmental regional observation system
CART	Classification and regression tree
CO	Carbon monoxide
CO${}_{2}$	Carbon dioxide
CV	Cross-validation
DRNN	Deep recurrent neural networks
DTT	Dynamic pre-training
EELM	Ensemble extreme learning machine
FC	Fight controller
FPV	First person view
FRP	Fiber reinforced plastics
GBR	Gradient boosting regression
GBM	Gradient boosting machine
GPS	Global positioning system
GPIO	General-purpose input–output
GT	Ground truth
GUI	Graphical user interface
I2C	Inter-integrated circuit
IoT	Internet of things
IMU	Inertial measurement unit
INP	Ice nucleation particles
*k*-NN	*k*-nearest neighbor
LCS	Long-range wireless communication system
LiDAR	Light detection and ranging
LoRa	Long range
LPWA	Low power wide area
LPWAN	Low-power wide-area network
LSTM	Long short-term memory
MAE	Mean absolute error
MSS	Multi-sensor system
NBIoT	Narrow band-Internet of things
NDIR	Non-dispersive infrared
NO	Nitric oxide
NO${}_{2}$	Nitrogen dioxide
OS	Operating system
PM	Particulate matter
PP	Polypropylene
PWM	Pulse width modulation
RF	Random forest
RM	Receiver module
RNN	Recurrent neural network
RTC	Real-time clock
RVS	Real-time visualization system
SBC	Single board computer
SfM	Structure from motion
SGD	Stochastic gradient descent
SVR	support vector regression
SVM	Support vector machine
TEOM	Tapered element oscillating microbalance
TM	Transmitter module
3D	Three-dimensional
UAV	Unmanned aerial vehicles
UFP	Ultrafine particles
USB	Universal serial bus
UV	Ultraviolet
